# Clinical Features of Hypersensitivity Pneumonitis in Children: A Single Center Study

**DOI:** 10.3389/fped.2021.789183

**Published:** 2022-01-20

**Authors:** Feizhou Zhang, Tongyu Yang, Zhixuan Liu, Xuan Jia, Li Yang, Lei Wu, Lanfang Tang

**Affiliations:** ^1^Department of Pulmonology, National Clinical Research Center for Child Health, Zhejiang University School of Medicine, The Children's Hospital, Hangzhou, China; ^2^Wenzhou Medical University, Wenzhou, China; ^3^Department of Radiology, National Clinical Research Center for Child Health, Zhejiang University School of Medicine, The Children's Hospital, Hangzhou, China

**Keywords:** hypersensitivity pneumonia, interstitial lung disease, antigen, glucocorticoid, children

## Abstract

**Background:**

Hypersensitivity pneumonia (HP) is an interstitial lung disease (ILD) mainly involving small airways and lung parenchyma that is caused by the inhalation of antigens in susceptible people to stimulate the body's immune response.

**Methods:**

A total of 6 Chinese children with HP treated in our center from July 2017 to July 2021 were included in our study.

**Results:**

Among the children, there were 4 males and 2 females, ranging in age from 4 to 14 years. Three cases had chest tightness and shortness of breath, 2 cases had cough, 1 case had chest pain, and 1 case had fever. Two cases of children had a history of close contact with pet dogs, 1 case had a history of contact with pigeons, 2 cases lived in a moldy house recently, and 1 case recently played a saxophone that had been idle for more than 2 years. The parents of two cases also had similar symptoms recently. The specific signs of chest HRCT of 6 cases all were in line with the characteristics of HP. After avoiding the sensitization environment, 2 children quickly recovered, 4 patients received low-dose glucocorticoid oral treatment, and after symptom control the dose was gradually reduced. The course of treatment was about 3–6 months.

**Conclusions:**

Exposure to a potential antigen has been found in all 6 HP children. The clinical manifestations are heterogeneous and easy to confuse with other diseases. A clear history of exposure to the antigens, respiratory symptoms associated with HP, signs of HP on HRCT, and improvement after removal from the antigenic environment constitute the cornerstone of the diagnosis of HP children in our unit. Avoiding exposure to antigenic environment is the first step in treatment, and glucocorticoid use is necessary in children with persistent symptoms.

## Background

Hypersensitivity pneumonitis (HP), also known as extrinsic allergic alveolitis, refers to a combination of immune complex-mediated (type III) and cell-mediated/delayed (type IV) hypersensitivity reactions to inhaled antigens (1). HP is a rare disease that can occur in people of all ages, with an average diagnosis age of 50–60 years (2). However, the number of HP diagnoses in children is far fewer than in adults. Due to the different causes and intensity of exposures, differences in geographical area, and differences in local climate and customs, there are no accurate epidemiological data of HP in the world (3). In children beyond the age of 2 years, more than half of all new cases of interstitial lung diseases (ILDs) in Germany were due to HP (4). Studies reviewing lung biopsies of pediatric patients with ILD have shown that HP can be present even in children younger than 2 years of age. Generally, the most common ILD entity diagnosed in children who have positive environmental exposure history is HP. HP is also one of most common diagnoses in children with ILD who are immunocompetent without systemic disorders (5). The latest HP guidelines divide HP into acute HP and chronic HP according to the course of the disease, and non-fibrotic HP and fibrotic HP according to histological characteristics, which have different clinical, imaging, and histological features (6). The clinical manifestations of HP patients are heterogeneous and are affected by various factors, such as the type of antigens, exposure time, timing of treatment, and genetics. The diagnosis and treatment guidelines for HP have been improved with the efforts of researchers and tend to be comprehensive (7).

Herein, we analyzed clinical characteristics, treatment, and outcome of HP children in our center for the purpose of improving clinicians' understanding of this disease.

## Materials and Methods

### Criteria for Diagnosis

We refer to the HP diagnostic criteria published in 2016. Acute HP can be diagnosed if main characteristics are fulfilled. If all of these main features are not fulfilled, one of substituted characteristics can function (Table 1).

**Table 1:** Proposed diagnostic criteria of acute HP (8).

**Main characteristics**	**Substituted characteristics**
Exposure to a potentially offending antigen source	Bronchoalveolar lavage lymphocytosis
Recurrent episodes of symptoms, occurring 4–8 h after exposure	Pathology of lung specimen consistent with acute HP
Elevated titer of specific IgG (precipitating) antibodies to an antigen	Positive laboratory inhalation challenge test (ICT), positive workplace challenge, or improvement after avoidance of the suspected exposure
Inspiratory crackles on physical examination	
HRCT pattern compatible with acute or subacute HP	

### Patients

Our study included children who were clinically diagnosed as HP in the Children's Hospital of Zhejiang University School of Medicine from July 2017 to July 2021. Clinical data, including previous history, birth history, family history, clinical manifestations, biochemical indicators, chest HRCT, diagnosis and treatment, and outcome, were, respectively, collected and analyzed in detail.

This study was approved by the ethics committee of the children's hospital affiliated to Zhejiang University School of Medicine and informed consent was obtained from the children's parents.

### Bronchoscopy and Bronchoalveolar Lavage (BALF)

After admission, the chest HRCT of the six children showed ILD, which was consistent with the indication of bronchoscopy. The specific process is as follows. First, relevant preoperative examinations should be improved, such as blood routine examination, blood coagulation function, and emergency immunization. Second, the purpose of bronchoscopy, possible complications during operation, and anesthesia should be explained to parents or guardians, and the informed consent should be signed. The child is then given general anesthesia by an anesthesiologist and first aid medications and monitoring equipment are prepared. Finally, children's bronchoscopy was performed by a respiratory endoscopist. The endoscopist injects 37°C normal saline (1 ml/kg/time, ≤ 20 ml/time, total ≤ 5–10 ml/kg), then passes the negative pressure 100–200 mmHg (1 mmHg = 0.133 kPa; the selected negative pressure value is used for the bronchial cavity during suction and it is advisable not to collapse) aspirator to obtain BALF; the recovery rate of each BALF should be ≥40%. A cytocentrifugal smear device was used, then spare cell suspension was added, centrifuged at 1,200 r/min for 10 min at 4°C, and a certain number of BALF cells were spread directly on the slide by centrifugation and downloaded. The slides are immediately blown dry with cold air, placed in absolute ethanol and fixed for 30 min, then stained with H&E. A total of 200 cells were counted under a 40 × optical microscope, and cytological classification was performed. Blood oxygen saturation and ECG continued to be monitored after surgery for the presence of dyspnea, hemoptysis, fever, etc. Fasting and water prohibition were observed for 2 h after surgery. According to the condition of the child, oxygen inhalation and sputum aspiration could be continued to keep the respiratory tract smooth, and special attention should be paid to the presence of fever, hemoptysis, pneumothorax, and other complications.

### Pulmonary Function Tests

Our center purchased a new lung function diagnosis system called MasterScreen from CareFusion, Germany. To determine the type of respiratory dysfunction and the severity of the disease in HP children, we performed lung function tests for them. First, the height and weight of the child were measured, and then the child was asked to pinch his nose, breathe through his mouth, and cooperate with the doctor to perform exhalation and inhalation. The time is about 30–40 min. We perform pulmonary function test and analysis in accordance with “Standardization of Spirometry 2019 Update. An Official American Thoracic Society and European Respiratory Society Technical Statement” (9).

### High-Resolution CT of the Chest

The chest HRCT images of six children were from the same model of equipment (GE Optima 660MSCT). The scanning parameters are set as follows: 100 kVp; auto mA, NI8 10; layer thickness, 5 mm. The scanning range starts from the entrance of the thorax and goes down to the bottom surface of the lung. Children under 5 years old need to take 10% chloral hydrate orally or enema (0.5 ml/kg, the total amount does not exceed 10 ml) before sedation.

All HRCT images were independently read by two experienced pediatric thoracic radiologists from our hospital, and the HRCT images were strictly recorded (using the indicators reported in the literature for the performance of adult HP). The cases that cannot reach a consensus were read together, and finally a unified result was obtained. The clinical and pathological data of the cases were concealed to radiologists, as well as other information such as the gender and age of the children.

### Statistical Analysis

The qualitative variables are described in terms of the number of participants (percentage) using Microsoft Office Excel software 2019.

## Results

### Patient Information

The specific information of the 6 children is shown in Table 2. It showed that there were 2 females, 4 males, all 6 children with ages over 2 years, and 4 children with allergic rhinitis or sinusitis. It was worth noting that the family history was similar to the children with symptoms in case 3'mother and in case 4'father. There were 2 cases of cough, 3 cases of chest tightness, 3 cases of shortness of breath, and 1 case each of chest pain or fever. Regarding the exposure source, 2 cases were close contact with pet dogs, 1 case was close contact with pigeons, the floor of 2 cases home was moldy, and 1 case played an idle saxophone. However, we are unable to determine the concentration, specific duration, and specific components of these children exposed to antigens.

**Table 2:** HP children information.

**Case**	**Gender**	**Age (years)**	**Weight (kg)**	**Medical history**	**Family history**	**Chief complaint**	**Exposure**
1	M	4	20	Eczema, urticaria, and sinusitis	Rhinitis	Cough	Pet dog
2	M	10	30.5	Sinusitis	None	Chest pain	Pet dog
3	F	10	34	Allergic rhinitis	Asthma	Chest distress	Pigeon
4	F	10	31.8	None	HP	Chest distress and shortness of breath	Moldy house
5	M	14	74.5	None	None	Chest distress and shortness of breath	Idle saxophone
6	M	13	50	Allergic rhinitis	None	Cough, shortness of breath, and fever	Moldy house

### Physical Examination

The highest body temperature of 1 case with fever was 39.4°C, and the breathing rate of 3 cases with shortness of breath was faster than that of normal children of the same age and sex, and the corresponding blood oxygen saturation was lower than normal (Table 3). Only 1 case had wet rales through lung auscultation; the rest were crackles. Examination of the heart, abdomen, and nervous system showed no obvious abnormalities.

**Table 3:** Physical examination of HP children.

**Case**	**T (**°**C)**	**R (times/min)**	**P (times/min)**	**SpO_**2**_ (%)**	**HP (mmHg)**	**Lung**
1	36.8	28	114	98	98/63	Wet rales
2	36.4	30	118	98	122/71	Crackles
3	36.6	24	96	97	112/65	Crackles
4	37.4	36	106	87	107/60	Crackles
5	36.8	32	74	92	105/57	Crackles
6	39.4	35	76	85	125/68	Crackles

### Laboratory Findings

We entered part of the peripheral blood test items into Table 4. It can be observed that the IgE level of 6 cases was significantly increased, and the eosinophil count of 3 cases was significantly increased, but the parasites, fungus (1-3)-β-D-glucan quantitative test, galactomannan, *Aspergillus fumigatus* IgG, M antibody quantitative test, glucuronoxylomannan, *Mycobacterium tuberculosis*, and respiratory viruses (adenovirus, influenza virus A+B, parainfluenza virus, syncytial virus, etc.) were all negative.

**Table 4:** Laboratory findings of HP children.

**PB items (normal range)**	**Case 1**	**Case 2**	**Case 3**	**Case 4**	**Case 5**	**Case 6**
Ig G (5–10.6 g/L)	7.36	7.9	10.6	6.15	8.5	11.8
Ig A (0.34–1.38 g/L)	0.96	0.8	1.34	0.75	0.6	2.23
Ig M (0.44–1.44 g/L)	1.3	1.14	1.87	1.2	1.4	2.88
Ig E (0–100 IU/ml)	1,160	674	897	956	784	688
C3 (0.5–1.5 g/L)	1.53	0.846	1.11	0.7	0.95	1.284
C4 (0.1–0.4 g/L)	0.29	0.251	0.36	0.21	0.32	0.376
Eosinophils (1–6%)	52.4	23.3	52.2	3.1	2.9	1.8
CD19 (23–30%)	16.71	15	18.43	17.65	15.42	36.45
CD3 (56–67%)	67.5	73.3	87.43	75.32	78.65	57.45
CD4 (33–39%)	22.69	26.45	25.41	30.16	23.67	24.75
CD8 (16–24%)	40.14	32.5	35.85	44.54	38.76	25.55
CD3–CD16+CD56+ (8–16%)	10.08	7.2	11.23	12.5	7.8	7.6
CD4/CD8 (1.6–2.2/1)	0.57	0.81	0.71	0.68	0.61	0.97

### Chest HRCT

**Figure 1:**
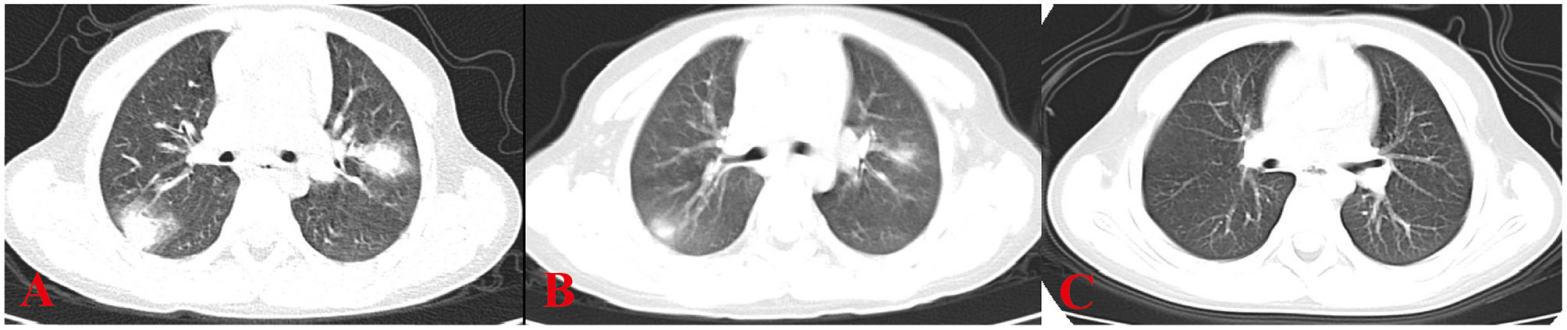
HRCT sign changes of case 1. **(A)** Multiple masses of ground glass–like increased density can be seen close to the subpleural of the outer lung field, the center density was much higher than the periphery, and the periphery showed a halo sign or faint cloudiness. **(B)** After 1 week of treatment, the high-density shadows of both lungs are absorbed than before. **(C)** After 2 weeks of treatment, the peripheral lesions are absorbed before the central lesions and are finally completely absorbed without leaving any traces of the lesions.

**Figure 2:**
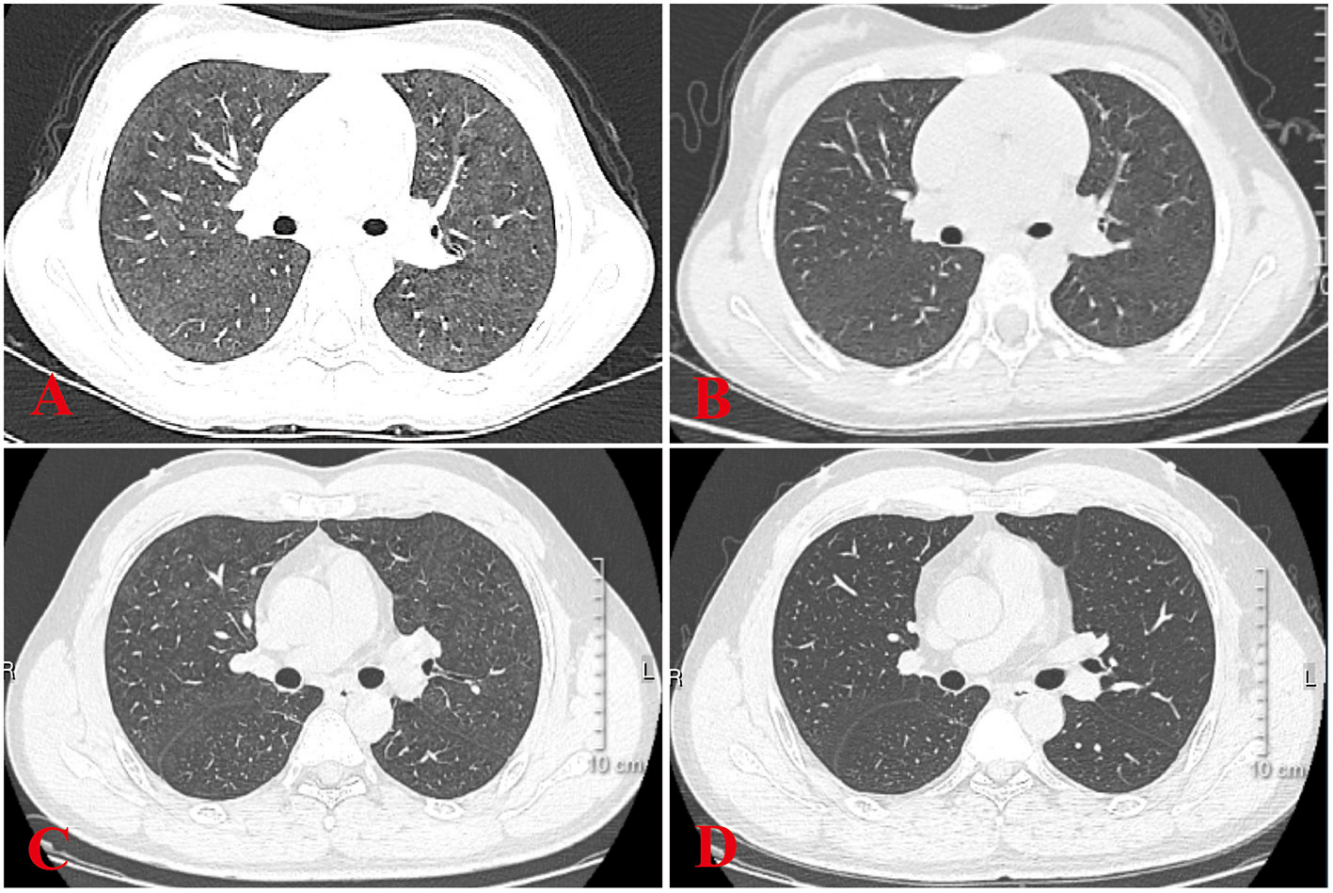
HRCT sign changes of case 4 and her father. **(A)** Ground glass shadows can be seen in both her lungs. **(B)** After 2 weeks of treatment, small subpleural nodules in the lower lobe of the left lung can be seen, and there were no obvious abnormalities in the remaining lung field. **(C)** Her father's chest signs were similar to hers when diagnosed as HP at a local hospital. **(D)** After 1 month of treatment, there were no obvious signs of abnormality in the lung.

All 6 children with HP underwent HRCT. Although they all met the signs of ILD, the specific signs were different (Figures 1, 2). We and two radiologists (Dr. Jia and Dr. Yang) conducted a detailed analysis and summary of the HRCT of 6 children with HP, as shown in Table 5. The lesions of 3 children with HP were diffusely distributed in both lungs, and 3 cases were distributed close to the subpleural of the outer lung field. Six cases showed ground-glass sign on different levels, 1 case had typical mosaic pattern, and 3 cases had nodules. Soft-tissue mass was seen in 1 case, and swollen lymph node was seen in 2 cases. No pleural effusion was seen in all children.

**Table 5:** HRCT features of HP children.

**Lung window**	**Mediastinal window**
Ground-glass opacities (6/6)	Soft tissue mass (1/6)
Centrilobular nodules (3/6)	Swollen lymph nodes (2/6)
A mosaic pattern (1/6)	Pleural effusion (0/6)
Head-cheese sign (3/6)	
Diffuse distribution (3/6)	
Close to the subpleural of the outer lung field (3/6)	

### Bronchoscopy and BALF

Three cases underwent bronchoalveolar lavage. The BALF contained mainly macrophages, and lymphocytes accounted for 6, 6, and 3%, respectively, and there was no significant increase.

### Pulmonary Function Tests

All 6 cases underwent pulmonary function tests, suggesting mild restrictive ventilatory dysfunction and negative diastolic test (Table 6).

**Table 6:** Pulmonary function test results of HP children.

**Pulmonary function test**		**Case 1**	**Case 2**	**Case 3**	**Case 4**	**Case 5**	**Case 6**
Before treatment	FEV_1_	74.7	73.8	74.6	75.6	74.9	75.1
	FVC	73	72.2	73.4	74.7	73.6	74
	FEV_1_/FVC	102.3	102.2	101.6	101.2	101.8	101.5
	PEF	81.8	80.4	81.4	82.6	82.1	82
	MMEF 75/25%	71.6	70.3	71.3	72.8	71.9	72
After treatment	FEV_1_	82.5	83.7	82.3	85.1	82.8	84.6
	FVC	81.3	82.4	81.7	84.3	81.1	83.5
	FEV_1_/FVC	101.5	101.6	100.7	100.9	102.1	101.3
	PEF	83.4	82.8	83.9	83.7	84	83.6
	MMEF 75/25%	75	73.5	75.4	76.1	75.2	76.3

*FEV1, forced expiratory volume in 1 s; FVC, forced vital capacity; PEF, peak expiratory flow; MMEF, maximum mid-expiratory flow*.

### Treatment and Prognosis

The symptoms of 2 cases were relieved after they were separated from the original exposure, and 4 cases received glucocorticoid (1 mg/kg/day) treatment for 3–6 months. When the symptoms are obvious and during the hospitalization, the glucocorticoids are infused intravenously. When the symptoms are controlled, we change the adrenal glucocorticoids to oral. In addition, for HP children with shortness of breath, oxygen was given in the acute phase. Re-examination of chest HRCT and lung function in 6 children showed no obvious abnormalities.

## Discussion

In summary, we retrospectively reviewed and analyzed the clinical data of 6 children with HP admitted to our center. The 6 children received the acute diagnosis and treatment plan, and the prognosis was satisfactory.

It was previously reported that as of 2005, 95 children with HP had been reported worldwide (10). However, due to the narrow age range, limited environmental exposure, and non-specific clinical and imaging features, these may be related to a variety of other infections. Furthermore, immune-mediated diffuse lung diseases overlap, so HP is likely to be underreported and underdiagnosed in the pediatric population (11). Due to lack of prior knowledge, we believe that the number of children with HP in China is far underestimated. Most HP are sporadic; however, recent studies have found the existence of familial HP, which mainly occurs in Japan (12). Among our 6 children, two of the children's patients also suffered from HP at the same time, but due to economic factors, genetic testing was not performed. Studies have found that gene mutations related to telomere shortening, such as TERT, TERC, RTEL1, and PARN, are related to the pathogenesis of HP (13). In addition, further studies surface that MHC class II regions, proteins involved in antigen processing and presentation, and immune proteasome components will increase the risk of HP (14), and the increased risk of chronic HP is related to the MUC5B promoter polymorphism rs35705950 (15). This reminds researchers that research on genome-wide associations to assess genetic susceptibility is necessary. The specific pathogenesis of HP in children needs further study by multiple medical centers.

HP is an immune-mediated ILD that occurs in genetically susceptible individuals after repeated inhalation of organic antigens (such as fungi, bacteria, and animal and insect proteins), which may lead to an immune response to acute lung inflammation (16). It is characterized by recruitment of granulocytes as well as T cells and B cells (17). This immune response can become chronic, leading to the activation of myofibroblasts and the deposition of extracellular matrix (18). In certain individuals with progressive factors (such as aging, further exposure, or genetic susceptibility), key pathogenic changes occur, leading to the expansion and activation of fibroblasts and myofibroblast populations, and excessive extracellular matrix accumulate and destroy the lung structure, eventually leading to lung fibrosis (19). However, for children, once HP is diagnosed, most will return to normal soon after leaving the sensitized environment and rarely progress to fibrotic HP. This also suggests that lung biopsy is unnecessary (20). Children with HP in our center have been traced back to a clear history of sensitization. It is worth noting that in addition to pet dogs, pigeons, and moldy houses, more and more children are now showing interest in musical instruments, so before using wind instruments, thorough cleaning and disinfection are necessary to attract the attention of parents and children. This is similar to previously reported potential causes for musicians playing contaminated wind instruments as HP (21).

Patients with acute and subacute HP may have non-specific manifestations, such as fever, cough, shortness of breath, wheezing, and even dyspnea. In patients with chronic HP, the main manifestations are weight loss, decreased activity endurance, and small blisters on lung auscultation (22). Surprisingly, most of the children diagnosed in our center start with chest tightness, except for other system abnormalities. Combined with the history of exposure to the antigenic environment, it is necessary to be highly vigilant against the occurrence of HP.

For the diagnosis of HP, in addition to the medical history and specific pathogen IgG detection to identify antigens, other related tests, such as HRCT, BALF cytology analysis, and lung biopsy histology, mainly play a diagnostic role, while respiratory pathogen testing, parasite screening, special pathogen testing, etc. mainly play a role in differential diagnosis. The detection of specific antigen IgG is suspicious because it cannot be determined whether the increase in IgG is caused by contact with the antigen or by the body's immune response after exposure. In addition to testing for respiratory viruses, we also tested for fungi, cryptococcus, *Aspergillus fumigatus*, and parasites, and the results were all negative. Therefore, we believe that it is difficult to identify specific pathogens in children. In addition, the significant increase in eosinophil counts in the peripheral blood of 3 children aroused our attention. Except for parasitic infections, the significance of the increase in eosinophil counts in children with HP needs to be explored. In the past decade, circulating biomarkers related to HP, such as lung epithelial-derived proteins (KL-6 (23), YKL-40 (24) and CCL17 (25)) and ANA (26), have been well-studied. Six children with HP were tested for anti-nuclear antibodies in our center, and the results were all negative, and the conditions for testing other markers are temporarily unavailable.

Six children received HRCT after admission. Compared with HP chest HRCT features in the 2020 HP guidelines, interstitial changes in small airways with nodules or masses, rather than large-scale consolidations, were observed in HP children. However, HP children's chest HRCT in our center has some different characteristics. There were 3 cases of children with HRCT that showed increased mass shadows before treatment, a center density that was much higher than the periphery, and the periphery showing a halo sign or faint cloudiness. The lesion is close to the outer band of both lungs and close to the pleura. When it is treated, it appears that the peripheral lesions are absorbed before the central lesions and are finally completely absorbed without leaving any traces of the lesions. The HRCT signs of children with HP in our center are different from ILDs, such as *Aspergillus* infection, pulmonary hemorrhage, and pulmonary alveolar proteinosis. This should arouse the attention of imaging physicians to strengthen cooperation with clinicians and to provide an extra layer of insurance for the diagnosis of children with HP. Recently, one case report referred that lung FDG uptake, or its combination with CT findings, may be a biomarker for predicting disease severity or progression from subacute to chronic HP (27).

Since its introduction in the 1970s, bronchoalveolar lavage (BAL) has become increasingly recognized as a low-risk research tool that can provide potential prognostic value (28). When combining BALF cell analysis with HRCT and experienced multidisciplinary discussions, it may help narrow the differential diagnosis and help avoid surgical lung biopsy (29). In the characteristic BALF of HP, lymphocytosis (usually >50%) is less pronounced in chronic fibrotic HP, but is usually at a higher level than other ILDs. Some researchers maintain that BALF lymphocytosis >30% can distinguish chronic fibrosis HP from idiopathic pulmonary fibrosis. BALF cell morphology, such as T-cell activation and foamy macrophages, can also be used to support the diagnosis of HP (30). The clinical utility of a reduced CD4/CD8 ratio (usually between 0.5 and 1.5 in HP) is controversial because it varies greatly and often does not decrease or even increase in chronic HP (31). To our surprise, there was no significant increase in lymphocyte counts in the BALF of 3 children who underwent bronchoscopy. Due to insufficient knowledge, CD4/CD8 was not tested in our center. For children with HP, chronic fibrotic HP is relatively rare, and whether it progresses to pulmonary fibrosis needs to be carefully considered. In addition, whether the guardian is willing to accept the bronchoscopy of the child to obtain the results of the examination also needs to be considered, and the proficiency of BAL also has a certain influence on the results.

All 6 children completed the pulmonary function examinations with a high degree of cooperation, suggesting restrictive ventilation dysfunction, which is consistent with the changes in pulmonary function caused by small airway disease, but it is of no diagnostic value because many other ILDs can also cause the same changes. However, as a non-invasive test, lung function plays a certain role in evaluating the recovery process of children with HP.

The treatment principle of HP is to remove the exposure source, oral systemic corticosteroids, and immunosuppressive therapy (32). For chronic fibrotic HP, anti-fibrotic therapy is necessary (33). If children's HP is not effectively treated in the acute phase, it may become chronic HP. Among the 6 cases, 2 cases improved rapidly after breaking away from the exposure source, and 4 cases received glucocorticoid therapy, and the treatment effect was satisfactory. Other treatment options, such as the application of immunosuppressants, biological inhibitors, and anti-fibrosis drugs, have been reported; however, the center has no relevant clinical experience yet.

## Conclusions

This study is the first national center in China to conduct a study on HP in children. First, it emphasizes the importance of finding a clear history of exposure and at the same time getting rid of pathogenic factors as early as possible is the first measure of treatment. Second, multi-disciplinary discussions facilitate the diagnosis of HP in children, such as the wisdom of the imaging department, the strategizing of the respiratory department, and the full assistance of the laboratory department. Finally, after more than 20 years of development, BAL has played an increasingly important role in the diagnosis and treatment of ILD in children. We will carry out more comprehensive and meaningful research on BALF in the near future for children with HP to provide more clues.

## Data Availability Statement

The raw data supporting the conclusions of this article will be made available by the authors, without undue reservation.

## Ethics Statement

This study was approved by the Ethics Committee of the children's hospital affiliated to Zhejiang university school of medicine and informed consent was obtained from the child's parents. Written informed consent to participate in this study was provided by the participants' legal guardian/next of kin. Written informed consent was obtained from the individual(s), and minor(s)' legal guardian/next of kin, for the publication of any potentially identifiable images or data included in this article.

## Author Contributions

FZ completed the first draft. TY, ZL, XJ, LY, and LW have participated in the data collection and improved the later revision of the article. LT revised the article to ensure the authenticity and practicability. All authors approved the final article as submitted and agree to be accountable for all aspects of the work.

## Funding

This work was supported by National Natural Science Foundation of China (81170016, 81470214, and 82070028) and Zhejiang Provincial Program for the Cultivation of High-Level Innovative Health Talents (2016).

## Conflict of Interest

The authors declare that the research was conducted in the absence of any commercial or financial relationships that could be construed as a potential conflict of interest.

## Publisher's Note

All claims expressed in this article are solely those of the authors and do not necessarily represent those of their affiliated organizations, or those of the publisher, the editors and the reviewers. Any product that may be evaluated in this article, or claim that may be made by its manufacturer, is not guaranteed or endorsed by the publisher.

## Abbreviations

HP: Hypersensitivity pneumonitis
ILD: Interstitial lung disease
HRCT: High-resolution CT
BAL: Bronchoalveolar lavage
BALF: Bronchoalveolar lavage fluid.
